# Toxicity of perfluorinated carboxylic acids for aquatic organisms

**DOI:** 10.2478/v10102-010-0014-2

**Published:** 2010-06

**Authors:** Miloň Tichý, Radka Valigurová, Radomír Čabala, Rut Uzlová, Marián Rucki

**Affiliations:** 1 Laboratory of Predictive Toxicology, National Institute of Public Health, Praha, Czech Republic; 2 Charles University in Prague, Faculty of Science, Department of Analytical Chemistry, Praha, Czech Republic

**Keywords:** perfluorinated carboxylic acids, *Tubifex tubifex*, water pollution, effective exposure time, acute toxicity

## Abstract

Toxicity of perfluorinated carboxylic acids with carbon chain C_8_ to C_12_ were tested with oligochaeta *Tubifex tubifex*. Toxicity was evaluated as the exposure time ET_50_ from onset of damage of the oligochaeta in saturated aqueous solutions. The ET_50_ fluctuated between 25 and 257 minutes. No statistically significant difference was found among the C_8_, C_9_ and C_12_ acids (ET_50_ between 143 and 257 minutes with large standard deviation). The acids with carbon chain C_10_ and C_11_ induced the effect significantly quicker (25 to 47 minutes). No acute toxicity measured in the three-minute test was observed in any case.

## Introduction

It is impossible to mention all the fluorinated products used nowadays in miscellaneous human activities, whereas naturally occurring ones are more than rare. Moreover, fluorinated derivatives of organic compounds belong to the most stable compounds, badly degradable, easily distributed in various media and matrices over the world. Their unique chemical properties make them important ingredients in various products. The compounds are found as widespread environmental contaminants. The global-scale multispecies mass balance model was used for simulation of the long-term transport of polyfluorinated carboxylic acids containing 8 to 13 carbons (Armitage *et al*., 2009). Model scenarios estimated their direct emission and indicated that the mass fluxes to the Arctic marine environment associated with oceanic transport were in excess of mass fluxes from indirect sources (atmospheric transport of precursors such as fluorotelomer alcohols and subsequent degradation to perfluorinated carboxylic acids, PFCAs).

Organic fluorinated acids are present in both arctic and antarctic ice or in equatorial waters. The polyfluoroalkyl acids and, in the past, fluoro-chloro methanes or ethanes belong among the most extended ones. The fluoro-halogen alkanes were used in sprays, their ethers as anesthetics, perfluorooctanoic and perfluorooctane sulphonate acids are widely used today in a lot of products as repellents, paper coatings, coatings for leather, upholstery or carpets, in fire fighting foams, pharmaceutics, insecticides, *etc*., (Alloatti *et al*., 2008, Houde *et al*., 2006, Kovarova & Svobodova, 2008).

On the other hand, monofluorinated organic compounds are important in clinical disciplines. Thus *e.g.* during the last decades, 2-deoxy-2-fluoro-D-glucose has been used to assess glucose uptake in the normal and diseased heart muscle (Hariharan *et al*., 1995), fluoro-derivatives of benzimidazole-quinoline were found to be inhibitors of the multitargeted receptor tyrosine kinase and on aspiration to be an anticancer drug (Henk *et al*., 2005), or fluoro-derivatives of 1,4-benzoquinone were used for studying the participation of ubiquinone in bovine heart mitochondrial respiratory chain, among many other products. The introduction of fluorine atoms into biologically active compounds proved to be beneficial. Several antitumor agents of this type can be named, such as fluorouracil, floxuridin, tegafur, getitinib or camptothecin (Alloatti *et al*., 2008).

And all these compounds eventually end up in our environment.

Generally, acute toxicity of polyfluorinated compounds is low (Kovarova and Svobodova, 2008), yet there is a lack of toxicological data. There is also a lack of data on their physico-chemical properties. Due to discrepancies in measurements performed by different methods, confidence in the existing data is low. Data on physicochemical properties should therefore be used as estimates only (Hertzke *et al*., 2007). It can be concluded that QA/QC should be carefully considered when generating and interpreting the results of analyses on both toxicological and physicochemical data. Often, uncommon properties of polyfluorinated organic compounds are a result of their structure, which resembles a rigid rod.

The aim of this contribution is a proposal how to estimate the relative toxicity of polyfluorinated compounds. Acute toxicity determination using oligochaeta *Tubifex tubifex* and the relevant mode of approach have been thoroughly described (Tichy & Rucki, 1996, Tichy *et al*., 2007; 2008). The duration of action of polyfluorinated organic compounds in oligochaeta *Tubifex tubifex* was taken as the basis for this consideration.

## Material and methods

### Determination of ET_50_

The perfluorinated acids C_8_ (95%), C_9_ (97%), C_10_ (98%), C_11_ (95%) and C_12_ (95%) were obtained from Aldrich (Steilheim, Germany). Always fresh oligochaeta *Tubifex tubifex* were purchased in a common shop. Manganese chloride (extra pure grade, Merck) was used as a reference compound (Tichy *et al*., 2007) to check the quality of the test object.

Solutions of C_8_ to C_12_ linear perfluorinated acids were prepared and tested at the concentration of 400 µg/ml (from 0.44 × 10^–3^ mol/l for C_12_ to 0.58 × 10^–3^ mol/l for C_8_), which was close to saturated concentrations. The amount of exactly 0.01 g of acid was weighed and dissolved by 15 ml water in a 25 ml plastic volumetric flask and sonicated 10 min at room temperature (22–24°C). The resulting aqueous acidic solution (pH 3 to 4) was neutralized by titration with a solution of sodium hydroxide (0.1 mol/l) to about pH 7 (checked by pH-meter) and filled up to 25 ml. Neutralization of the solutions was necessary because the low acidity itself could affect the oligochaeta.

ET_n_ is the effective time when n % of individuals from the population exposed shows a recognizable reaction. In our case, ET_5_, ET_50_ and ET_95_ were determined by counting the number of immobilized *Tubifex tubifex* during the exposure time. Immobilized worms were counted each half hour for five hours of exposure at room temperature (22–24°C). Deformation of bodies of the oligochaeta was observed: they wrinkled, bled and finally the worms were destroyed and broke up into pieces. Thus immobilization could be noticed only with difficulties. Each solution was tested three times in various seasons and always in a triplicate. The experimental data were processed with special computer software (Logistic Method for Determination of LD_50_ by National Institute of Public Health, Prague) modified for calculation of ET_50_.

## Results

At the concentration of 400 µg/ml, aqueous solutions of perfluorinated aliphatic carboxylic acid are not toxic if the acute toxicity is measured in three minutes with *Tubifex tubifex* (Tichý *et al*., 1996; 2007). The three-minute exposure caused no apparent changes of *Tubifex tubifex.* Thus the solutions of the acids were not acutely toxic for the oligochaeta and the common testing procedure could not be applied.

However, the effect begins to be noticeable after a longer time, in chronic exposure, and the time necessary for induction of observable changes could be taken as a measure of toxicity of the acids. The start of the effect depends on the chain length of the given acid, however with the minimum at C_11_ acid.

The results are summarized in Table 1, which contains values and confidence intervals (n = 3, α = 0.05) of ET_5_, ET_50_ and ET_95_ for the time necessary to immobilize 5%, 50% and 95%, respectively, of the oligochaeta from the whole population under exposure. The minimum ET_50_ value appeared with perfluoroundecanoic acid (C_11_) being comparable with perfluorodecanoic acid (C_10_).

**Table 1:** Effective time ET_50_, ET_5_ and ET_95_ (mins.). *Tubifex tubifex* was exposed in neutral aqueous saturated solutions of perfluorinated carboxylic acids (C_8_–C_12_).

Perfluorinated acid	Effective time (min)		

	ET_5_	ET_50_	ET_95_
**Octanoic**			
C_7_F_15_COOH	111 (62–141)	274 (240–347)	675 (471–1687)
**Nonanoic**			
C_8_F_17_COOH	155 (106–181)	284 (257–339)	519 (405–972)
**Decanoic**			
C_9_F_19_COOH	23 (12–32)	59 (47–70)	151 (118–233)
**Undecanoic**			
C_10_F_21_COOH	13 (7–18)	32 (25–39)	74 (56–130)
**Dodecanoic**			
C_11_F_23_COOH	13 (7–18)	176 (143–221)	1073 (627–3328)

values in brackets represent confidence intervals (n = 3, α = 0.05)

## Discussion

We applied an uncommon measure of toxicity, ET_50_, the time necessary for the onset of destruction of the oligochaeta body. The shorter the ET_50_, the more toxic is a compound considered. We used this measure because no acute toxicity was observed, a fact that corresponded with the generally accepted opinion that perfluorinated carboxylic acids have limited toxicity for aquatic organisms.

Using the ET_50_ measure, the most toxic of the set of the compounds tested, i.e. linear polyfluorinated carboxylic acids, were perfluorodecanoic (C_10_) and perfluoroundecanoic (C_11_) acids. No statistically significant differences between the toxic indices exist among C_8_, C_9_ and C_12_ acids. The C_10_ and C_11_ acids form a clear minimum, statistically significantly distinguished, and thus considered the most toxic ones in chronic exposures. Naturally, this conclusion applies only for our laboratory conditions in a closed system (on Petri dishes).

The question arises how does log*P*_(n-octanol–water)_ of these acids or their salts behave. Unfortunately, even with simpler alcohols and fluorotelomers, it is still an unsolved problem because of the unique behavior of the fluorine substituents in the compounds. As far as the image of perfluorinated acids as rigid rods is true, a bending or a break may exist at C_10_ and C_11_. We can thus just speculate that there is some non-linearity at the C_10_ to C_11_ carbon chain. The ability to penetrate membranes by this special form of compounds could then be influenced by this fact, although a better penetrating *Tubifex* surface with a rod is more probable than with anything else. The effect in the oligochaeta starts much sooner with C_10_ and C_11_ perfluorinated acids than with shorter or longer carbon chains.

## References

[CIT0001] Alloatti D, Giannini G, Cabri W, Lustrati I, Marzi M, Ciacci A, Gallo G, Tinti MO, Marcellini M, Riccioni T, Guglielmi P, Pisano C (2008). Synthesis and biological activity of fluorinated Combrestatin analogues. J. Med. Chem..

[CIT0002] Armitage JM, MacLead M, Cousins IT (2009). Comparative assessment of the global fate and transport pathways of long-chain perfluorocarboxylic acids (PFCAs) and perfluorocarboxalates (PFCs) emitted from direct sources. Environ. Sci. Technol..

[CIT0003] Herzke D, Schlabach M, Mariussen E, Uggerud H, Heimstad E (2007). A literature survey on selected chemical compounds.

[CIT0004] Broxterman HJ, Georgopapadakou NH (2005). Anticancer Therapeutics: “Addictive” multi-targeted drugs, new drug combinations. Drug Resistance Updates.

[CIT0005] Houde M, Martin JW, Letcher RJ, Solomon KR, Muir DCG (2006). Biological monitoring of polyfluoroalkyl substances: A review. Environmental Science & Technology.

[CIT0006] Kovarova J, Svobodova Z (2008). Perfluorinated compounds: occurrence and risk – A review. Neurotoxicol. Letters.

[CIT0007] Hariharan R, Bray M, Ganim R, Doenst T, Goodwin GW, Taegtmeyer H (1995). Fundamental limitations of [^18^F]2-deoxy-2-fluoro-D-glucose for assessing myocardial glucose uptake. Circulation.

[CIT0008] Tichý M, Rucki M (1996). An alternative method for determining acute toxicity of chemicals: cessation of movements in *Tubifex tubifex* worms. Pracov Lék.

[CIT0009] Tichý M, Rucki M, Hanzlíková I, Roth Z (2007). The *Tubifex* tubifex assay for the determibation of acute toxicity. ATLA.

[CIT0010] Tichý M, Rucki M, Hanzlíková I, Roth Z (2008). Acute toxicity determination by calculation – *Tubifex tubifex* assay and quantitative structure – activity relationships. Environ. Toxicol. Chem..

[CIT0011] Valigurova R (2008). Thesis: Toxicity determination of salts of perfluorinated organic acids, Charles University in Prague.

